# Spontaneous Hyphema during Ibrutinib Treatment in a CLL Patient

**DOI:** 10.1155/2023/1691996

**Published:** 2023-12-14

**Authors:** Kim Abbegail Tan Aldecoa, Chef Stan L. Macaraeg, Akash Dadlani, Sri Yadlapalli

**Affiliations:** ^1^Department of Internal Medicine, Trinity Health Oakland, Pontiac, MI, USA; ^2^Wayne State University, Detroit, MI, USA; ^3^University of Connecticut, Farmington, CT, USA; ^4^Ross University School of Medicine, Bridgetown, Barbados; ^5^Department of Hematology and Oncology, Trinity Health Oakland, Pontiac, MI, USA

## Abstract

Ibrutinib is an oral, first-line, targeted therapy for chronic lymphocytic leukemia (CLL). Commonly reported adverse events are diarrhea, fatigue, and musculoskeletal pain, but rarely it has been associated with visual disturbances. Here, we present a rare case of spontaneous hyphema in a 60-year-old patient with a known diagnosis of CLL on ibrutinib treatment.

## 1. Introduction

Targeted therapy for cancer treatment is becoming popular because of less toxic side effects than conventional chemotherapy. Ibrutinib is a medication that blocks the activity of a protein called Bruton's tyrosine kinase (BTK) and is primarily used as a first-line agent for treating chronic lymphocytic leukemia (CLL). It is also used for other disorders, including Waldenstrom's macroglobulinemia, and previously treated chronic graft versus host disease [1]. By binding irreversibly to BTK, which is essential for replication and spread in B cell cancers, ibrutinib prevents the downstream effects of BTK. In the product information for ibrutinib, adverse events were analyzed across clinical trials, revealing that among treated CLL patients, common side effects occurred in ≥30% of the cases. These include thrombocytopenia, diarrhea, fatigue, musculoskeletal pain, neutropenia, rash, anemia, bruising, and nausea [1]. Bleeding events, encompassing bruising and petechiae, were also prevalent, occurring in 39% of the cases, although the underlying mechanism remains unclear [1]. Visual disturbances have infrequently been reported, with limited case reports and case series available in the literature [2, 3]. The study aims to present a rare ocular side effect associated with ibrutinib.

## 2. Case Presentation

**Figure 1:**
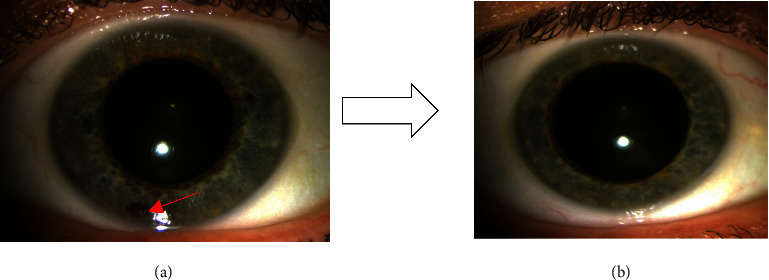
Left eye showing (a) 1 × 1 mm blood clot on presentation (red arrow) and (b) resolution on a two-week follow-up.

The patient is a 60-year-old Caucasian woman with CLL, on ibrutinib treatment 420 mg daily for four months. Her past medical history included hypothyroidism on levothyroxine and chronic complex regional pain syndrome on medical marijuana. She has no family history of blood disorders. She presented to the emergency department (ED) with a 1-day history of visual disturbance described as a “smudge on her glasses.” She denied any pain or trauma to the eye. Physical examination was significant for left eye hyphema at the 6 o'clock position (Figure 1(a)). There were no other obvious signs of bruises or bleeding noted. Complete blood count (platelet count 189,000/*μ*L), prothrombin time, and activated partial thromboplastin time were unremarkable. Computed tomography of the head showed intact globes without conspicuous intraocular hemorrhage or foreign body. Ophthalmology was consulted. Her visual acuity was 20/20 on the right eye and 20/25 on the left eye. Tonometry in the ED showed intraocular pressure of 4–6 mmHg in the left eye and 5–7 mmHg in the right eye. A Seidel test with a fluorescein dye was also carried out, which did not reveal any corneal or scleral defect. Oncology was consulted, and ibrutinib was discontinued. She was discharged on prednisolone 1% four times daily, timolol 0.25% twice daily, and cyclopentolate hydrochloride 1% eye drops twice daily.

On a two-day follow-up with the ophthalmologist, the patient is still complaining of intermittent blurring of vision, but it has improved. A slit lamp exam revealed a 1 × 1 mm blood clot at the 6 o'clock position of the anterior chamber of the left eye. Fundus examination, tonometry (12 mmHg right eye and 13 mmHg left eye), pupillary exam, and visual fields were unremarkable. She was advised to continue using prednisolone 1% eye drops for the next two weeks. Timolol was discontinued.

Symptoms resolved completely at a two-week follow-up (Figure 1(b)). Although there was no life-threatening reaction, after careful consideration and discussion with the patient, a decision was made to switch to different chemotherapeutic agents (bendamustine and rituximab) due to concerns about the potential recurrence of hyphema. Fortunately, there were no further occurrences of hyphema following the change in medication.

## 3. Discussion

Hyphema is a condition where there is blood collection in the eye's anterior chamber from disruption of the vessels of the iris or ciliary body. The most common cause is blunt trauma, but it can occur spontaneously or after minor trauma in patients with bleeding tendencies [4]. Risk factors for spontaneous hyphema include neovascularization from uncontrolled diabetes mellitus, clotting disorders, medications that act as anticoagulants, or those that inhibit platelet function [4]. None of these are present in our patient.

Ibrutinib generally has a safe ophthalmologic profile, although there have been increasing isolated ocular toxicities reported recently. This includes red or dry eyes, blurred vision, subconjunctival hemorrhage, uveitis, branch retinal artery occlusion, and cystoid macular edema, and the development of cataracts has been identified [2, 5–9].  Table 1 summarizes the ocular side effects reported in a few case studies.

The reason for ocular disturbances associated with ibrutinib is still unclear, but several mechanisms have been suggested. One case report showed a patient experiencing a spontaneous hyphema, which Bohn and colleagues attributed to ibrutinib's deficiency in platelet adhesion [7]. Levade et al. found in vitro evidence that ibrutinib reduces platelet adhesion to von Willebrand factor and platelet aggregation induced by collagen [11], supporting this hypothesis. Compared to other BTK inhibitors, ibrutinib was reported to carry a higher risk of bleeding due to its angiogenesis inhibition and stronger cytotoxic effect on endothelial cells [12]. It is worth noting that our patient's platelet counts and bleeding parameters were normal.

In addition, uveitis is a more common visual disturbance reported with ibrutinib, as shown in Table 1. This could be due to ibrutinib's off-target effect on mitogen-activated protein kinase kinase (MEK) protein, which is responsible for protecting the eyelids, conjunctiva, and cornea. The inhibition of MEK protein may lead to inflammation and uveitis by dysregulation of tight junctions of the endothelial cells of the ciliary body, which may contribute to inflammation leading to uveitis [3, 13, 14]. Furthermore, the inhibition of these protein kinases has been shown to trigger a Th1 proinflammatory response and autoimmune phenomenon [2]. Other kinase inhibitors have also been reported to cause nonspecific visual toxicities, such as anaplastic lymphoma kinase (ALK) inhibitors, epidermal growth factor receptor (EGFR) inhibitors, MEK inhibitors, FMS-like tyrosine kinase 3 (FLT3) inhibitors, and other tyrosine kinase inhibitors, including ibrutinib and acalabrutinib [2, 3].

Since the mechanism is still unclear for visual disturbances associated with ibrutinib, treatment has been limited to case reports and case series. Most patients have complete resolution of symptoms upon discontinuation of ibrutinib and topical steroid eye drops (as shown in Table 1), similar to our patient. Some case studies have reported changing to different chemotherapeutic agents after discontinuation of ibrutinib [7, 9], while others have restarted ibrutinib after their symptoms have resolved [2, 8].

## 4. Learning Points/Conclusion

The resolution of the patient's hyphema upon discontinuation of ibrutinib treatment suggests a causative link. Our research highlights the occurrence of spontaneous hyphema as another potential ocular toxicity of ibrutinib. As targeted immunotherapies become more common in the treatment of hematologic malignancies, it is important to remain vigilant for such ocular toxicities as they may become more common in the future. A more comprehensive understanding of the cellular and biochemical processes associated with these treatments is still necessary as the mechanism is still unclear. Lastly, it is essential to be aware of possible ocular toxicities associated with this first-line immunotherapy and to discontinue the drug early, if necessary, to prevent catastrophic consequences.

## Figures and Tables

**Table 1:** Summary of ocular findings observed with ibrutinib.

Reference	Age (years)	Duration of ibrutinib treatment	Initial symptoms	Ophthalmologic findings	Treatment	Outcome
Bohn et al. [7]	65	1 day	Bilateral blurring of vision	Uveitis	Ibrutinib discontinued; significant improvement after local and oral steroids	Clinical improvement
Bohn et al. [7]	64	9 months	1-week blurring of vision and photophobia, with 3-day hyphema	Uveitis	Responded poorly with local steroids while continuing ibrutinib and with significant improvement when ibrutinib was discontinued	Clinical improvement
Chiu et al. [2]	60	1 year	Bilateral increase in floaters	Uveitis	Ibrutinib continued; resolved with topical steroids	Clinical improvement
Chiu et al. [2]	63	2 years	Left eye floaters	Uveitis and cystoid macular edema	Ibrutinib continued; responded well with topical and oral steroids initially, but with relapses of uveitis with cystoid macular edema	Clinical improvement, but with relapses
Chiu et al. [2]	69	1 year and 6 months	Right eye loss of vision and floaters	Uveitis	Ibrutinib was discontinued temporarily but restarted after a trial of topical and IV steroids	Clinical improvement
Chiu et al. [2]	66	3 years	3-week history of bilateral floaters	Uveitis	Ibrutinib continued; responded well with topical steroids	Clinical improvement
Saenz-de-Viteri and Cudrnak [8]	67	4 weeks	4-week history of bilateral blurry vision	Cystoid macular edema	Ibrutinib continued at a normal dose; responded well with topical steroids and NSAIDS	Clinical improvement
Ben-Avi et al. [9]	73	Over 3 years	Left eye decreased in vision	Cystoid macular edema	Ibrutinib was discontinued; responded well with topical steroids and NSAIDS	Clinical improvement
Kolomeyer et al. [10]	75	6 months	Blurring of vision after cataract surgery	Anterior chamber fibrinoid syndrome after cataract extraction	Unknown if ibrutinib was continued; responded well with topical steroids after 2 weeks	Clinical improvement

## Data Availability

Additional information for this study is available upon reasonable request.

## Abbreviations

BTK: Bruton's tyrosine kinase
CLL: Chronic lymphocytic leukemia
ED: Emergency department
MEK: Mitogen-activated protein kinase kinase
ALK: Anaplastic lymphoma kinase
FLT3: FMS-like tyrosine kinase 3.
